# Milk fat globule membrane plus milk fat increase docosahexaenoic acid availability in infant formulas

**DOI:** 10.1007/s00394-022-03024-5

**Published:** 2022-10-25

**Authors:** Antonio Gázquez, María Sabater-Molina, Inés Domínguez-López, María Sánchez-Campillo, Neus Torrento, Joan Tibau, José A. Moreno-Muñoz, María Rodríguez-Palmero, María C. López-Sabater, Elvira Larqué

**Affiliations:** 1 Department of Physiology, University of Murcia, Campus Mare Nostrum, Murcia, Spain; 2 Biomedical Research Institute of Murcia (IMIB-Arrixaca), Murcia, Spain; 3 Department of Nutrition, Food Sciences and Gastronomy, Faculty of Pharmacy and Food Sciences, University of Barcelona, Barcelona, Spain; 4 Institute of Agrifood Research and Technology (IRTA), Monells, Barcelona, Spain; 5 Laboratorios Ordesa SL, Barcelona, Spain

**Keywords:** DHA, Infant formula, Milk fat globule membrane, Gut, Villous

## Abstract

**Purpose:**

Milk fat globule membrane (MFGM) has components with emulsifier properties that could affect the provision of substrates to the brain. We evaluated the effects of MFGM plus milk fat addition to infant formulas on docosahexaenoic acid (DHA) availability and gut development.

**Methods:**

In Experiment 1, suckling piglets were divided into 3 groups: Group L1 (*n* = 8): fed with a vegetal fat formula with palm oil; L2 (*n* = 8): canola oil formula and L3 (*n* = 8): milk fat + canola oil + 1% Lacprodan (3% MFGM of total protein content). In Experiment 2, Group L4 (*n* = 7): fed with canola oil + 1% Lacprodan (3% MFGM) and Group L5 (*n* = 5): milk fat + canola oil + 2% Lacprodan (6% MFGM). All formulas contained 0.2% DHA and 0.2% arachidonic acid.

**Results:**

In Experiment 1, DHA was similar among the groups in both total fatty acids and plasma phospholipids (PL). However, 3% MFGM (L3) increased significantly the proportion of DHA and LC-PUFA *n*-3 in liver total fatty acids, jejunum, and also in jejunum PL respect to the other formulas. There were no changes in gut histology, cell proliferation, apoptosis, or brain DHA content. In Experiment 2, higher MFGM dose was used. Then, higher DHA was not only found in peripheral tissues of 6% MFGM (L5) piglets but also in plasma PL, while a similar trend was observed in cortex PL (*p* = 0.123).

**Conclusion:**

In conclusion, MFGM plus milk fat may increase DHA availability of infant formulas which could contribute to their beneficial health effects.

**Supplementary Information:**

The online version contains supplementary material available at 10.1007/s00394-022-03024-5.

## Introduction

Improving the nutritional and health properties of infant milk formula by the addition of functional ingredients is of primary importance. Milk fat globule membrane (MFGM) enriched formulations may now be used in vegetable oil-based infant formulas to provide polar lipids and bioactive compounds. The MFGM consists of a bilayer of polar lipids, primarily phosphatidylcholine (PC), sphingomyelin (SM), cholesterol as well as cerebrosides, gangliosides, glycosylated proteins and polypeptides, filaments, mucins, lactadherin, butyrophilin, and others [1]. Polar lipids account for about 1% of milk fat [2]. In the last decade, the importance of milk phospholipids (PL) and of the MFGM in early life nutrition has been reported (e.g., for infant growth, brain and cognitive development, gut microbiome and maturation, and defense against infections) [3–7]. Potential health benefits of MFGM may be related to choline, ethanolamine, and polyunsaturated fatty acid (PUFA) effects on the gut mucosa [8], inhibitory effect on cholesterol absorption, or to an influence on the gut microbiome and immune function [9].

In addition, human milk supplies the neonate with long-chain polyunsaturated fatty acids (LC-PUFAs), being arachidoinc acid (ARA, 20:4 *n*-6) and docosahexaenoic acid (DHA, 22:6 *n*-3) of special importance for newborn growth and development. They occur predominantly in the triacylglycerol (TAG) form and to a lesser extent as part of milk MFGM (PL form). The MFGM provides physical properties to the milk fat globule that enhances TAG digestion [2]. The access of the gut to milk polar lipids and water-soluble choline and ethanolamine favors mucosal and hepatic PC and phosphatidyl-ethanolamine (PE) generation. ARA from milk PL and TAG may be selectively retained in mucosal PL and transported with chylomicron PC, PE, and chylomicron remnants, as reported in a study with stable isotopes [10]. This favors the uptake of ARA by the liver and this could also occur for other PUFAs as DHA. The metabolism of chylomicron PLs and TAGs after feeding milk fat may contribute to higher PUFAs gut uptake which might influence the beneficial effects of MFGM on neurodevelopment and other issues. While mechanisms of action remain unclear, MFGM may have direct effects on the brain (e.g., provision of substrates for brain structural development), indirect effects via gut development (e.g., promotion of intestinal epithelial cell proliferation and changes in microbial colonization), and the gut–brain axis. MFGM narrows the gap between breastmilk and formula-fed infants/pups in many aspects related to growth, intestinal differentiation/maturation, brain development, and cognition [3–7]; although more studies are needed to better understand the mechanisms behind and the long-term health implications of its supplementation.

The aim of this study was to evaluate the effects of infant formulas elaborated with different fat blends and enriched with bioactive compounds (MFGM and milk fat) on DHA availability and histological gut structure in suckling piglets. All the assayed formulas contained the same amount of both ARA and DHA.

## Materials and methods

### Study design

#### Experiment 1

Twenty-four Iberian piglets crossbreeding PI*(DU*LS) of 5 days old were stabulated in the farm from the Institute of Agrifood Research and Technology (IRTA) of Monells (Girona, Spain). They were randomly assigned to three experimental groups (*n* = 8/group). Piglets from each group were housed together and manually bottle-fed for 2–3 days; later on, they received the formula in a feeder three times per day. Heating was provided with spot heat lamps attached above each cage. This study was approved by the Animal Care Committee at the IRTA (CEEA 038/2017, N.9648) and conforms the European Union Regulation of Animal Care for the care and use of animals for research.

Three powdered formulas, identical in all ingredients except for fat composition, were designed to meet the nutrient requirement of growing piglets according to The National Research Council (NRC) nutrient requirements of pigs (Table 1). Piglets were litter-matched in all experimental groups to ensure a similar basal status of the animals at the entry of the study. The formulas were dissolved in warm water at a concentration of 20%. The piglets’ formulas were elaborated by Ordesa (Barcelona, Spain) with the following fat composition: Group L1 (*n* = 8): dairy formula in which fat was comprised by a standard mixture of vegetal fats designed for infant formula (containing palm oil), plus DHA and ARA, each at 0.2% of total fatty acids; Group L2 (*n* = 8): dairy formula with the same composition of L1 but the lipid fraction was replaced by canola oil (without palm oil) plus DHA and ARA (0.2% each of total fatty acids); Group L3 (*n* = 8): dairy formula with lipid fraction comprised by milk fat (butteroil with 60% of total lipids), canola oil, DHA and ARA (0.2% each of total fatty acids) and 1% Lacprodan MFMG-10 (Arla Ingredients, Denmark) which represented 3% total protein content of the formula and a concentration of 2 g/l. Lacprodan MFGM-10 bovine whey protein concentrate composition (g/100 g): protein 72 ± 2 g, lactose max. 2 g, sialic acid 1.5 g, total lipids 18 ± 2 g, phospholipids 6 ± 1 g (sphingomyelin 1.8 g, phosphatidyl-ethanolamine 1.5 g, phosphatidyl choline 1.7 g, phosphatidyl inositol 0.3 g, phosphatidyl serine 0.3 g, gangliosides 0.13 g, others phospholipids 0.5 g), cholesterol 0.6 g, and moisture max. 5 g. The three formulas provided the same amount of total energy, macronutrients, and micronutrients. The fatty acid composition of experimental formulas is reported in Table 2. The piglets were fed with an increasing volume of formula throughout the experiment depending upon animal weight (from 70 to 500 ml per day) with an average volume of 90 ml/kg/day. This provided an average intake of DHA and ARA about 5 mg/kg/day in all the groups and 0.2 g/kg/day of MFGM.

**Table 1:** Ingredient composition of experimental formulas

Ingredients	Experiment 1			Experiment 2	
(per 100 g powder)*	L1	L2	L3	L4	L5
Energy (kcal)	446.3	446.3	450.9	450.9	455.5
Protein (g)	22.5	22.5	23.2	23.2	23.9
Total fat (g)	16	16	16.2	16.2	16.4
Milk fat (g)	–	–	8	–	8
Lactose (g)	53	53	53	53	53
Fiber (g)	0.15	0.15	0.15	0.15	0.15
Choline (g)	0.21	0.21	0.21	0.21	0.21
Lacprodan MFGM-10 (g)	–	–	1	1	2
Minerals					
Na (mg)	453	453	453	453	453
Ca (mg)	901	901	901	901	901
P (mg)	546	546	546	546	546
Cl (mg)	1369	1369	1369	1369	1369
Fe (mg)	15	15	15	15	15
I (µg)	111.7	111.7	111.7	111.7	111.7
Mn (mg)	0.49	0.49	0.49	0.49	0.49
Zn (mg)	10.8	10.8	10.8	10.8	10.8
Se (µg)	15	15	15	15	15
Cu (mg)	1.95	1.95	1.95	1.95	1.95
Mg (mg)	106	106	106	106	106
Vitamins					
Vitamin A (µg)	1267	1267	1267	1267	1267
Vitamin D3 (µg)	22.5	22.5	22.5	22.5	22.5
Vitamin E (mg)	12	12	12	12	12
Vitamin B1 (µg)	369	369	369	369	369
Vitamin B2 (riboflavin) (µg)	1650	1650	1650	1650	1650
Vitamin B5 (pantothenic acid) (µg)	3605	3605	3605	3605	3605
Vitamin B6 (piridoxin) (µg)	700	700	700	700	700
Vitamin B9 (folic acid) (µg)	125	125	125	125	125
Vitamin B12 (µg)	13.5	13.5	13.5	13.5	13.5
Vitamin K (µg)	600	600	600	600	600
Biotin (µg)	36	36	36	36	36
Niacin (µg)	7420	7420	7420	7420	7420
Ascorbic acid (mg)	7.5	7.5	7.5	7.5	7.5

All the formulas contained 0.2% arachidonic acid (20:4 *n*-6) + 0.2% docosahexaenoic acid (22:6 *n*-3)

^*^Dissolved in warm water at a concentration of 20% (w/vol)

*L1* vegetal fat (with palm oil), *L2* Canola Oil, *L3* Canola Oil + milk fat + 3% of protein MFGM, *L4* Canola oil + 3% of protein MFGM, *L5* Canola oil + milk fat + 6% of protein MFGM

**Table 2:** Fatty acid composition of experimental formulas

Selected fatty acids	Experiment 1			Experiment 2	
(g/100 g)	L1	L2	L3	L4	L5
4:0	tr	tr	1.19	0.02	1.07
6:0	tr	tr	0.93	0.01	0.78
8:0	0.99	tr	0.61	0.01	1.36
10:0	0.79	tr	1.42	0.02	1.55
12:0	6.91	tr	1.74	0.03	6.69
14:0	3.26	tr	5.75	0.10	5.82
16:0	24.71	4.08	18.46	4.31	13.02
18:0	3.59	1.82	5.78	1.90	5.25
18:1 *n*-9	39.56	62.26	42.46	61.79	42.81
18:2 *n*-6	15.03	19.77	10.65	19.60	12.71
18:3 *n*-3	1.68	8.49	4.25	8.40	1.46
22:1 *n*-9	0.10	1.88	0.90	1.85	0.00
20:4 *n*-6	0.21	0.21	0.21	0.22	0.22
20:5 *n*-3	0.07	0.07	0.07	0.07	0.07
22:6 *n*-3	0.20	0.20	0.20	0.20	0.20

Only selected fatty acids are listed, but all fatty acids analyzed were considered for the calculation of percentages. Tr., traces

All the formulas contained 0.2% arachidonic acid (20:4 *n*-6) + 0.2% docosahexaenoic acid (22:6 *n*-3)

*L1* vegetal fat (with palm oil), *L2* Canola Oil, *L3* Canola Oil + milk fat + 3% of protein MFGM, *L4* Canola oil + 3% of protein MFGM, *L5* Canola oil + milk fat + 6% of protein MFGM

Piglets were sacrified at 21 days of age. The day before, piglets were deprived of food overnight and bled to death via jugular vein puncture while under anesthesia. Blood was collected in tripotassium EDTA-coated tubes (2.7 mmol/L) and centrifuged at 3000 × *g* for 10 min at 4 °C and stored at − 80 °C until analysis. Two pieces from liver and frontal cortex of brain were excised, washed in cool saline solution, dried with filter paper to remove excess water, frozen in liquid nitrogen, and stored at − 80 °C.

Three samples of gut tissue of 1 cm length at 10, 25, 75 and 90% of total length of small gut were collected in a histological casette under formol solution for histological analysis. Moreover, 250 mg of jejunum (around 25% position of gut length), ileum (around 75% of gut length), and colon were removed, rinsed thoroughly with cold saline solution, opened lengthwise, and blotted dry. The mucosa was removed by scraping the entire luminal surface with a glass coverslip over an ice-cold Petri dish and frozen in in liquid nitrogen and stored at − 80 °C.

### Experiment 2

Twelve Iberian piglets of 2–3 day age under the same conditions that in Experiment 1 were randomly assigned to two dietary groups. The formulas utilized in Experiment 2 were identical in all ingredients to those of Experiment 1 except for fat composition (Table 1): Group L4 (*n* = 7): dairy formula with canola oil, DHA, ARA (0.2% each of total fatty acids), and 1% Lacprodan MFMG-10 which represented 3% total protein content of the formula (2 g/l, and a daily intake of 0.2 g/kg/day of MFGM); Group L5 (*n* = 5): dairy formula with canola oil, DHA and ARA (0.2% each of total fatty acids), milk fat (butteroil), and 2% Lacprodan MFMG-10 which represented 6% total protein content of the formula (4 g/l, and a daily intake of 0.4 g/kg/day of MFGM). The fatty acid composition of experimental formulas is reported in Table 2. Piglets feeding regime and samples collected followed the same procedures described in Experiment 1.

### Fatty acid analysis

Fatty acid analysis from 100 mg of faeces was done according to the method of López-López et al. [11] using tridecanoic acid as internal standard. Fatty acids from plasma PL were isolated using 800 µl of plasma and Solid-Phase Extraction (SPE) in a 96-well plate (Discovery DSC-NH_2_, Supelco, Bellefonte, PA, USA). PL were eluted from SPE with 1.5 ml of methanol, methylated, and analyzed by gas chromatography [12].

Total lipids were also extracted from 0.2 ml pf plasma, 0.03 g of liver, 0.4 g of intestinal mucosa, and 0.04 g of cortex according to Folch et al. [13]. Previous to the extraction, an internal standard was added to the samples: 0.05 mg pentadecanoic acid for total fatty acid analyses, and 0.1 mg of each pentadecanoic acid, dipentadecanoyl phosphatidylcholine, tripentadecanoin, and cholesterylpentadecaoate for lipid fraction analyses. Samples with internal standard were extracted into chloroform: methanol (2:1 v/v) with butylhydroxytoluene (1 mg/ml) as antioxidant. The lipid extract was evaporated to dryness under nitrogen flux. The residue was taken up in 400 µL chloroform:methanol (1:1 v/v) for analysis of lipid fractions. It was applied on silica gel plates (Merck, Darmstadt, Germany) and the PL, TAG, cholesterol esters, and non-esterified fatty acids were isolated by development of the plates in n-heptane/diisopropylether/glacial acetic acid (60:40:3 v/v) [14]. Bands from different lipid fractions were detected with 0.2% 2′, 7′-dichlorofluorescein in ethanol (w/v) under UV light and scraped. The bands of the thin-layer chromatography as well as the dried residue for total fatty acid analyses were methylated according to Stoffel et al. [15] by adding 1 mL of 3 N methanolic HCl (Supelco, Sigma-Aldrich, MO, EEUU) and heating at 90 °C for 1 h. The derivatives were extracted into hexane and stored at − 20 °C until gas chromatographic analysis.

Fatty acid methyl esters were analyzed by gas chromatography using an SP-2560 capillary column (100 m × 0.25 mm × 20 µm) (Supelco, Sigma-Aldrich, MO, EEUU) in a Hewlett-Packard 6890 gas chromatograph (Agilent Technologies, CA, EEUU) equipped with a flame ionization detector [16]. The temperature of the detector and the injector was 240 °C. The oven temperature was programmed at 175 °C 30 min and increased at 2 °C/min to 230 °C and held at this temperature for 17 min. Helium was used as the carrier gas at a pressure of 45 psi. Peaks were identified by comparison of their retention times with appropriate fatty acid methyl esters standards (Sigma-Aldrich, MO, EEUU) and concentrations determined in relation to peak area of internal standard.

### Gut histology

The microscopical morphology measurements of the gut were performed with the five intestinal tissue samples collected at 10, 25, 50, 75 and 90% of the length of the small intestine. 10 and 25% refer to the jejunum region, while 50, 75 and 90% refer to the ileum. After fixation of the tissue samples in Bouin liquid (75 ml picric acid water saturated solution, 25 ml formaldehyde, and 5 ml acetic acid), a portion of each sample was embedded in paraffin wax using standard techniques. Two transverse sections were randomly selected from each sample, stained with hematoxylin and eosin, and examined with an optical microscope Axioskop (Zeiss, Germany). Villus length and crypt depth were measured using an image processing programme (Microm Image Processing, software 4.5). The length and depth of at least 10 villi and crypts were measured calculating both the mean villus length and the mean crypt depth. In addition, the number of cells per length of villus was estimated from 10 villi in each histological section [17, 18].

### Gut immunofluorescence

To evaluate gut cell proliferation and apoptosis, both caspase-3 and anti-ki-67 were quantified by immunofluorescence. After paraffin fixing, tissue samples were sliced into 5 μm sections and rehydrated. Antigen retrieval was performed by boiling the slides in citrate buffer. Nonspecific binding was blocked with 1% bovin serum albumin (Sigma-Aldrich, MO, EEUU) in phosphate-buffered saline at room temperature for 1 h. Samples were labelled with specific antibodies against active caspase-3 (Dako) or anti-ki-67 (Abcam) at 1:100 ratio with 0.1% bovin serum albumin in phosphate-buffered saline by overnight incubation at 4 °C. Furthermore, 2 h incubation with mouse secondary antibodies (AlexaFluor 568, A-11011, Thermo Fisher Scientific, MA, EEUU) in concentration 1:200 was performed at room temperature.

Cell nuclei were stained with Hoechst 33,342 at 10 μg/ml concentration for 30 s at room temperature (Life Technologies, CA, EEUU). Slides were mounted in Fluoromount Aqueous.

Mounting Medium (Sigma-Aldrich, MO, EEUU). Sequence scanning was used to omit cross-talk between fluorescent dyes. Confocal microscopy (Leica Microsystems, Barcelona, Spain) was employed for in-tissue cytometry analysis. Ten images per one tissue sample were made. Quantification of apoptotic cells was performed according to in-tissue cytometry procedure by measurement of active caspase-3 expression [19].

### Statistical analysis

Sample size was estimated based on DHA percentages in plasma PLs of piglets published by Alessandri et al. [20]. Type I error was set at *α* = 0.05 and type II error *β* = 0.2 (power 80%), obtaining a minimum sample size of three animals per group. The software used for this estimation was nQuery 7.0 (Statsols HQ, Cork, Ireland).

The SPSS 24.0 software (SPSS, Inc., IL, EEUU) was used for statistical analyses. Differences between groups were assessed by one way ANOVA followed by post hoc of Bonferroni in Experiment 1 and by T test in Experiment 2. Statistical significant differences were establish at *p* < 0.05. Data are expressed as means ± standard deviation.

## Results

### Experiment 1

In Experiment 1, three different formulas were tested: L1, a formula with a vegetal fat blend (containing palm oil), L2 with canola oil (without palm oil) and a formula with canola oil plus milk fat serum and 3% MFGM (L3). Formula L2, without palm oil, had the lowest palmitic acid (PA, 16:0) content that was mainly substituted with oleic acid (OA, 18:1 *n*-9) and essential fatty acids (linoleic acid (LA) 18:2 *n*-6 and α-linolenic acid (ALA) 18:3 *n*-3). L3 formula contained 3% MFGM and milk fat that was added on the fat source of the formula L2; milk fat included PA in beta position in its composition reducing the differences on PA among formulas. The composition of LC-PUFA (both DHA and ARA) was similar in all formulas (Table 2).

**Fig. 1:**
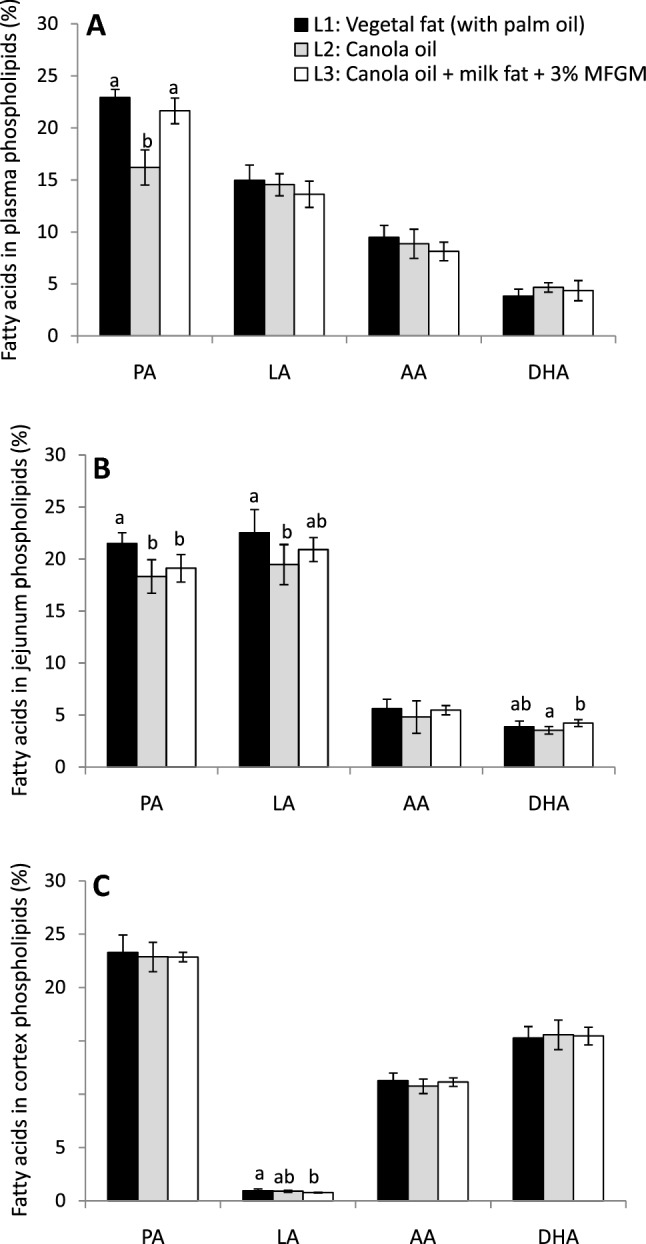
Fatty acid profile in **A** plasma phospholipids, **B** jejunum phospholipids, and **C** cortex phospholipids of infant formula-fed piglets with different fat composition: vegetal fat blend with palm oil (L1), vegetal fat with canola oil (without palm oil, L2), or with canola oil + milk fat serum + 3% MFGM (L3). The three formulas contained the same amount of DHA and AA (0.2% of total fatty acids). Data are means ± SD. N = 8/group. AA, arachidonic acid (20:4 *n*-6); DHA, docosahexaenoic acid (22:6 *n*-3); LA, linoleic acid (18:2 *n*-6); MFGM, milk fat globule membrane; PA, palmitic acid (16:0). Different letters indicate statistically significant differences between experimental groups (*p* < 0.05)

There were no differences in weight or growth among groups of piglets from Experiment 1 (L1: 3.55 ± 0.31 kg; L2: 3.46 ± 0.41 kg; L3: 3.30 ± 0.6 kg, *p* = 0.543). Concerning fatty acids in these animals, there were no differences in saturated fatty acids (SFA) or monounsaturated fatty acids (MUFA) in plasma total lipids between groups. Despite the big differences in PA composition among formulas, PA content in plasma total lipids of L2 was similar to that observed in the other groups, probably ameliorated by endogenous synthesis or lower PA uptake from faeces in L1 (Table 3). Nevertheless, in plasma PL, the differences according PA content among groups were more evident than in total lipids (Fig. 1A). DHA was similar among the groups in both total fatty acids of plasma (Table 3) and in plasma PL (Fig. 1A). Nevertheless, L2 (without palm oil) and L3 (with MFGM and milk fat) had higher proportion of total *n*-3 LC-PUFA in both total fatty acids (Table 3) and plasma PL (L1: 5.97 ± 0.75^a^, L2: 8.29 ± 0.84^b^ and L3: 7.93 ± 1.30^b^, *P* < 0.05) than L1.

**Table 3:** Fatty acid profile in total lipids of piglets from Experiment 1

Selected fatty acids		Plasma	Faeces	Jejunum	Liver	Cortex
(g/100 g)						
16:0	L1	**22.16 ± 0.77ª**	**52.58 ± 4.81**^**a**^	**21.82 ± 1.15ª**	**18.66 ± 0.68ª**	23.43 ± 1.81
	L2	**20.20 ± 2.63**^**ab**^	**21.61 ± 2.72**^**b**^	**18.06 ± 1.78**^**b**^	**13.14 ± 0.65**^**b**^	22.91 ± 0.62
	L3	**19.45 ± 2.26**^**b**^	**37.33 ± 3.79**^**c**^	**19.22 ± 1.35**^**b**^	**17.50 ± 0.91**^**c**^	23.00 ± 0.67
16:1 *n*-9	L1	0.52 ± 0.03	**0.46 ± 0.17**^**a**^	**0.48 ± 0.18**^**ab**^	0.48 ± 0.08	1.14 ± 0.09
	L2	0.54 ± 0.06	**1.14 ± 0.43**^**b**^	**0.60 ± 0.18ª**	0.50 ± 0.10	1.10 ± 0.08
	L3	0.55 ± 0.06	**0.66 ± 0.31**^**a**^	**0.38 ± 0.13**^**b**^	0.41 ± 0.06	1.12 ± 0.06
18:0	L1	12.23 ± 0.64	**26.98 ± 3.68**^**a**^	13.81 ± 1.63	18.97 ± 1.66	20.49 ± 1.85
	L2	12.78 ± 0.88	**45.67 ± 7.08**^**b**^	14.63 ± 1.96	19.89 ± 1.77	21.24 ± 0.51
	L3	12.57 ± 1.02	**35.96 ± 1.84**^**c**^	15.69 ± 1.84	19.36 ± 0.85	21.25 ± 0.29
18:1 *n*-9	L1	22.56 ± 0.73	**4.30 ± 0.70**^**ab**^	**21.64 ± 1.73ª**	**14.07 ± 1.86**^**ab**^	11.52 ± 0.92
	L2	22.79 ± 2.50	**3.44 ± 0.95**^**a**^	**25.16 ± 2.15**^**b**^	**15.76 ± 1.98ª**	11.12 ± 0.75
	L3	22.40 ± 0.62	**5.47 ± 1.70**^**b**^	**21.59 ± 1.22ª**	**13.32 ± 0.78**^**b**^	11.24 ± 0.71
18:2 *n*-6	L1	20.41 ± 1.62	**0.61 ± 0.15**^**a**^	**22.07 ± 2.33ª**	**14.01 ± 1.00**^**a**^	**1.06 ± 0.18ª**
	L2	19.63 ± 1.57	**0.33 ± 0.09**^**b**^	**19.77 ± 1.36**^**b**^	**14.58 ± 1.19ª**	**1.04 ± 0.10**^**ab**^
	L3	20.48 ± 1.46	**0.36 ± 0.11**^**b**^	**20.57 ± 0.76**^**ab**^	**12.69 ± 0.58**^**b**^	**0.89 ± 0.04**^**b**^
18:3 *n*-6	L1	**0.50 ± 0.18**^**ab**^	n.d	**0.12 ± 0.03ª**	0.33 ± 0.19	tr
	L2	**0.53 ± 0.09ª**	n.d	**0.08 ± 0.01**^**b**^	0.28 ± 0.06	tr
	L3	**0.30 ± 0.21**^**b**^	n.d	**0.12 ± 0.02ª**	0.28 ± 0.08	tr
18:3 *n*-3	L1	**1.07 ± 0.50**^**a**^	0.05 ± 0.01	**0.84 ± 0.07ª**	**0.40 ± 0.21ª**	tr
	L2	**1.87 ± 0.96**^**ab**^	0.05 ± 0.01	**2.39 ± 0.26**^**b**^	**1.58 ± 0.44**^**b**^	tr
	L3	**2.03 ± 0.55**^**b**^	0.07 ± 0.03	**2.28 ± 0.26**^**b**^	**0.92 ± 0.20**^**c**^	tr
20:3 *n*-6	L1	0.60 ± 0.07	tr	0.70 ± 0.18	**0.87 ± 0.18**^**ab**^	1.03 ± 0.34
	L2	0.59 ± 0.14	tr	0.65 ± 0.26	**0.73 ± 0.09ª**	1.10 ± 0.38
	L3	0.51 ± 0.10	tr	0.95 ± 0.30	**0.95 ± 0.19**^**b**^	1.01 ± 0.38
20:4 *n*-6	L1	**5.92 ± 0.90**^**ab**^	tr	5.58 ± 1.03	13.51 ± 0.94	11.3 ± 0.54
	L2	**5.72 ± 0.79ª**	tr	4.45 ± 1.08	12.49 ± 1.21	11.19 ± 0.54
	L3	**6.72 ± 0.71**^**b**^	tr	5.32 ± 0.67	12.81 ± 0.53	11.32 ± 0.27
20:5 *n*-3	L1	**0.69 ± 0.37ª**	tr	**0.52 ± 0.09ª**	**0.62 ± 0.11ª**	0.30 ± 0.07
	L2	**1.29 ± 0.59**^**b**^	tr	**0.89 ± 0.17**^**b**^	**1.97 ± 0.61**^**b**^	0.24 ± 0.05
	L3	**1.29 ± 0.28**^**b**^	tr	**1.10 ± 0.20**^**c**^	**1.73 ± 0.38**^**b**^	0.29 ± 0.05
22:4 *n*-6	L1	0.27 ± 0.08	0.08 ± 0.06	0.52 ± 0.21	**0.61 ± 0.11ª**	3.96 ± 0.49
	L2	0.24 ± 0.06	0.16 ± 0.09	0.40 ± 0.18	**0.34 ± 0.03**^**b**^	3.89 ± 0.39
	L3	0.26 ± 0.09	0.11 ± 0.04	0.34 ± 0.07	**0.47 ± 0.09**^**c**^	4.05 ± 0.32
22:5 *n*-6	L1	0.12 ± 0.08	tr	0.20 ± 0.02	**0.46 ± 0.04ª**	2.35 ± 0.53
	L2	0.11 ± 0.08	tr	0.14 ± 0.08	**0.20 ± 0.03**^**b**^	2.19 ± 0.26
	L3	0.07 ± 0.07	tr	0.19 ± 0.03	**0.42 ± 0.07ª**	2.21 ± 0.42
22:5 *n*-3	L1	0.91 ± 0.18	tr	0.70 ± 0.12	**1.49 ± 0.19ª**	0.29 ± 0.11
	L2	1.12 ± 0.20	tr	0.83 ± 0.26	**1.74 ± 0.28ª**	0.34 ± 0.07
	L3	0.94 ± 0.32	tr	0.90 ± 0.10	**2.14 ± 0.19**^**b**^	0.35 ± 0.07
22:6 *n*-3	L1	3.23 ± 0.53	0.30 ± 0.24	**3.75 ± 0.61**^**ab**^	**7.28 ± 0.62ª**	15.00 ± 1.22
	L2	3.76 ± 0.51	0.43 ± 0.31	**3.44 ± 0.39ª**	**8.16 ± 1.01**^**ab**^	16.05 ± 1.21
	L3	3.66 ± 0.74	0.28 ± 0.11	**4.09 ± 0.45**^**b**^	**8.73 ± 0.60**^**b**^	15.29 ± 1.14
SFA	L1	38.87 ± 1.31	**92.58 ± 0.84**^**a**^	**38.33 ± 0.83ª**	**41.53 ± 3.10ª**	45.60 ± 0.45
	L2	37.55 ± 3.67	**89.81 ± 1.16**^**b**^	**34.93 ± 1.41**^**b**^	**36.25 ± 1.60**^**b**^	45.55 ± 0.52
	L3	36.20 ± 2.13	**87.99 ± 2.95**^**b**^	**37.60 ± 0.58ª**	**40.39 ± 0.88ª**	45.80 ± 0.76
MUFA	L1	26.74 ± 0.94	**5.13 ± 0.67**^**a**^	**25.72 ± 2.62ª**	**17.90 ± 2.18ª**	18.39 ± 1.33
	L2	26.98 ± 2.82	**6.69 ± 0.67**^**b**^	**30.94 ± 2.29**^**b**^	**20.68 ± 2.52**^**b**^	17.82 ± 1.18
	L3	26.99 ± 0.65	**7.05 ± 1.63**^**b**^	**25.55 ± 1.57ª**	**17.36 ± 1.04ª**	18.06 ± 0.95
PUFA	L1	**34.14 ± 1.84ª**	2.10 ± 0.66	**35.68 ± 2.23**^**ab**^	**40.50 ± 1.63ª**	35.75 ± 1.05
	L2	**35.28 ± 1.54**^**ab**^	3.36 ± 0.92	**33.82 ± 1.30ª**	**42.97 ± 1.05**^**b**^	36.43 ± 1.16
	L3	**36.67 ± 2.17**^**b**^	2.43 ± 1.38	**36.37 ± 1.35**^**b**^	**41.95 ± 0.68**^**ab**^	35.86 ± 0.85
LC-PUFA *n*-6	L1	7.04 ± 0.96	**1.14 ± 0.44**^**a**^	7.49 ± 1.21	**15.98 ± 1.01ª**	18.67 ± 1.33
	L2	6.78 ± 0.95	**2.56 ± 0.64**^**b**^	6.19 ± 1.39	**14.34 ± 1.29**^**b**^	18.37 ± 0.51
	L3	7.78 ± 0.69	**1.72 ± 1.32**^**ab**^	7.11 ± 0.65	**15.03 ± 0.54**^**ab**^	18.60 ± 0.82
LC-PUFA *n*-3	L1	**4.84 ± 0.91**^**a**^	0.30 ± 0.24	**5.04 ± 0.65ª**	**9.40 ± 0.61ª**	15.60 ± 1.18
	L2	**6.18 ± 1.18**^**b**^	0.43 ± 0.31	**5.34 ± 0.64ª**	**11.96 ± 1.73**^**b**^	16.65 ± 1.16
	L3	**5.90 ± 1.10**^**ab**^	0.28 ± 0.11	**6.16 ± 0.58**^**b**^	**12.61 ± 1.00**^**b**^	15.94 ± 1.13
*n*-6/*n*-3	L1	5.00 ± 1.33	–	**5.08 ± 0.43ª**	**3.10 ± 0.17ª**	1.27 ± 0.14
	L2	3.64 ± 1.36	–	**3.37 ± 0.19**^**b**^	**2.18 ± 0.26**^**b**^	1.17 ± 0.09
	L3	3.72 ± 0.81	–	**3.30 ± 0.22**^**b**^	**2.08 ± 0.23**^**b**^	1.23 ± 0.13
Δ6 desaturase	L1	2.94 ± 0.85	–	3.24 ± 1.08	**6.19 ± 1.17**^**ab**^	99.81 ± 37.59
	L2	3.01 ± 0.86	–	3.32 ± 1.32	**5.06 ± 0.94**^**a**^	104.10 ± 29.72
	L3	2.49 ± 0.78	–	4.66 ± 1.56	**7.45 ± 1.34**^**b**^	111.89 ± 40.40

Data are mean ± SD. Only selected fatty acids are listed, but all fatty acids analyzed were considered for the percentage calculation

All the formulas contained 0.2% AA (20:4 *n*-6) + 0.2% DHA (22:6 *n*-3). n.d., not detected; tr., traces. Δ6 desaturase was calculated as the ratio between 20:3 *n*-6/18:2 *n*-6. *N* = 8 animals/group. Data not sharing the same superscript letters within a tissue indicate statistical differences between study groups (*P* < 0.05) (bold face)

*L1* vegetal fat (with palm oil), *L2* Canola Oil, *L3* Canola Oil + milk fat + 3% MFGM

**Fig. 2:**
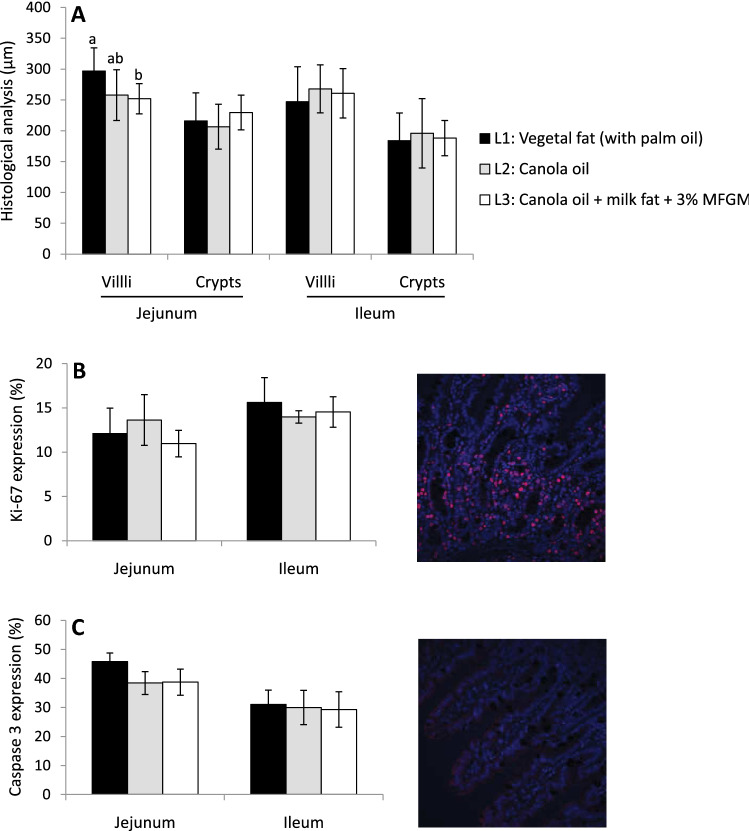
**A** Histological analysis parameters (villi length and crypts depth), **B** Ki-67 expression, and **C** Caspase 3 expression in gut samples (jejunum and ileum) of infant formula-fed piglets with different fat composition: vegetal fat blend with palm oil (L1), vegetal fat with canola oil (without palm oil, L2), or with canola oil + milk fat serum + 3% MFGM (L3). The three formulas contained the same amount of docosahexaenoic acid and arachidonic acid (0.2% of total fatty acids). Data are means ± SD. *N* = 8/group. Different letters indicate statistically significant differences between experimental groups (*p* < 0.05)

The higher LC-PUFA *n*-3 levels in plasma of Experiment 1 were not due to a better effect of formulas on gut structure, since the length of villi was lower in both L2 and L3 respect to L1 (Fig. 2A). In addition, there were no changes in crypth depth, cell proliferation (ki-67 expression), or apoptosis, evaluated by caspase-3 levels, in jejunum and ileum (Fig. 2B, C respectively). Moreover, there were no differences in the DHA or LC-PUFA *n*-3 content in faeces, while PA was higher in the faeces of the L1 followed by L3 and L2 (Table 3). However, 3% MFGM and milk fat of L3 increased significantly the proportion of both DHA and LC-PUFA *n*-3 in total fatty acids of jejunum (Table 3) and also in jejunum PL (Fig. 1B) with respect to the other formulas. Thus, the higher DHA levels in jejunum could be due to higher gut uptake driven by the polar lipids of MFGM plus milk fat.

We also explored the effect of MFGM plus milk fat addition to the formula in other tissues as liver or brain; we found significantly higher levels of DHA and LC-PUFA *n*-3 in liver of L3 and L2 with respect to the L1 (Table 3). Nevertheless, cortex fatty acid composition practically did not change among groups (Table 3, Fig. 1C). Thus, 3% MFGM plus milk fat increased DHA content in some tissues (jejunum and liver) but not in plasma or cortex in piglets of Experiment 1 (Table 3, Fig. 1).

### Experiment 2

In Experiment 2, the amount of MFGM was increased in the formulas and the animals were a bit younger than in Experiment 1. In the Experiment 2, the formula L4 contained a vegetal fat blend from canola oil plus 3% of the MFGM (without milk fat), while L5 piglets were fed with the same vegetal blend plus milk fat and 6% of the MFGM (both with similar DHA and ARA content than Experiment 1) (Table 2).

**Fig. 3:**
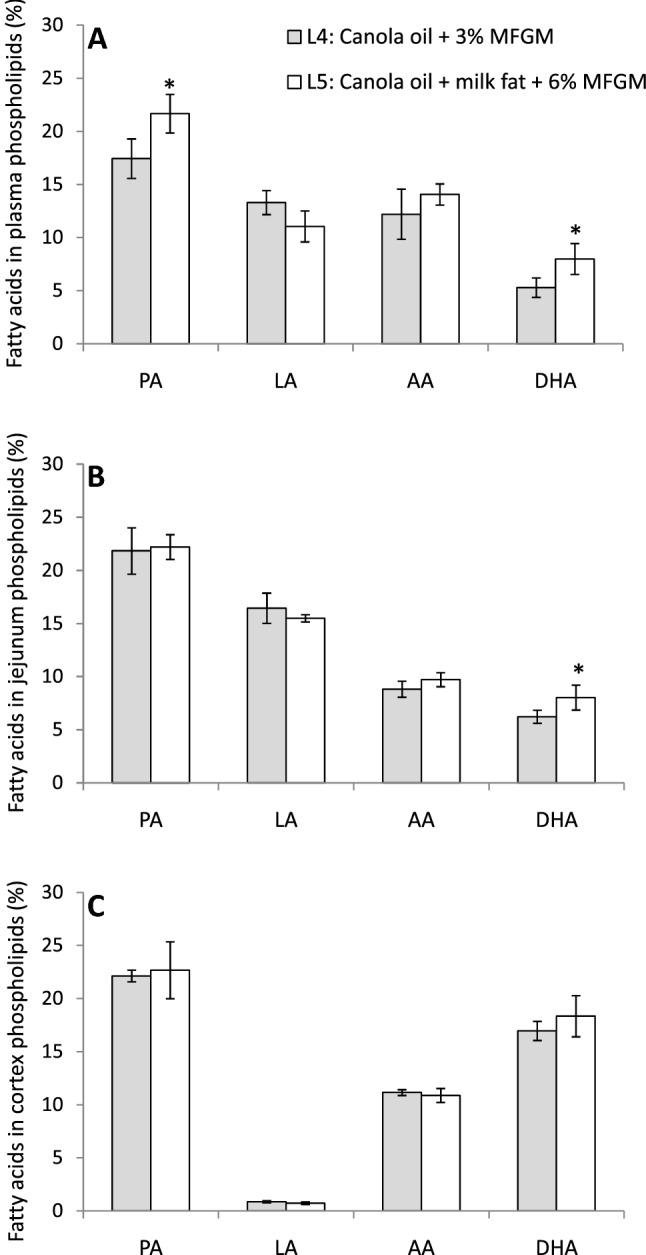
Fatty acid profile in **A** plasma phospholipids, **B** jejunum phospholipids, and **C** cortex phospholipids of infant formula-fed piglets with different fat composition: vegetal fat with canola oil (without palm oil) + 3% MFGM (L4) or with canola oil + milk fat serum + 6% MFGM (L5). The two formulas contained the same amount of DHA and AA (0.2% of total fatty acids). Data are means ± SD. L4 *n* = 7 and L5 *n* = 5. AA, arachidonic acid (20:4 *n*-6); DHA, docosahexaenoic acid (22:6 *n*-3); LA, linoleic acid (18:2 *n*-6); MFGM, milk fat globule membrane; PA, palmitic acid (16:0). *Indicates statistically significant differences between experimental groups (*p* < 0.05)

There were no differences in animal weight among groups from Experiment 2, although piglets from L5 tended to higher weight at the end of the experiment compared to those of L4 (L4: 3.15 ± 0.67 kg; L5: 4.55 ± 1.37 kg, *p* = 0.085). We found that adding milk fat serum plus 6% MFGM led to higher levels of DHA and PA in plasma PL (Fig. 3A). In addition, in the tissues, 6% MFGM also increased DHA content in PL of jejunum (Fig. 3B) and total fatty acids of liver (Table 4), and the same trend was observed in total fatty acids from jejunum (*p* = 0.068) and colon (*p* = 0.112) (Table 4). These differences in tissue DHA values could not be explain by differences in essential fatty acid composition of the formulas, since L5 (6% MFGM + milk fat) contained less LA and especially less ALA than formula L4 (3% MFGM) (Table 2). Moreover, Δ6 desaturase activity was similar in all tissues of both groups (Table 4). In the brain, 6% MFGM plus milk fat showed a slight trend towards higher DHA level in cortex PL (*p* = 0.123), although the differences were not statistically significant (Fig. 3C). Thus, 6% MFGM plus milk fat increased DHA not only in tissues (except brain) but also in plasma PL of the piglets.

**Table 4:** Fatty acid profile in total lipids of piglets from Experiment 2

Selected fatty acids (g/100 g)		Jejunum	Colon	Liver	Cortex
16:0	L4	18.47 ± 2.30	**17.09 ± 1.25**	**12.55 ± 1.93**	23.30 ± 0.74
	L5	20.80 ± 1.90	20.76 ± 0.33*	16.13 ± 0.35*	22.14 ± 1.84
16:1 *n*-9	L4	0.72 ± 0.17	**1.00 ± 0.07**	0.57 ± 0.20	**1.05 ± 0.06**
	L5	0.57 ± 0.11	0.78 ± 0.07*	0.49 ± 0.16	0.82 ± 0.13*
18:0	L4	12.22 ± 1.64	9.44 ± 1.94	17.75 ± 2.18	20.79 ± 0.70
	L5	11.25 ± 1.34	10.21 ± 0.78	17.83 ± 1.75	20.02 ± 2.35
18:1 *n*-9	L4	21.21 ± 4.56	31.34 ± 4.38	15.34 ± 2.39	**11.28 ± 0.55**
	L5	21.73 ± 4.12	31.82 ± 4.55	14.52 ± 3.83	13.55 ± 1.81*
18:2 *n*-6	L4	**18.25 ± 0.71**	**12.79 ± 1.86**	**12.36 ± 1.24**	0.91 ± 0.13
	L5	15.79 ± 0.19*	9.86 ± 0.24*	9.78 ± 0.66*	0.80 ± 0.14
18:3 *n*-6	L4	0.09 ± 0.04	0.14 ± 0.05	0.40 ± 0.14	tr
	L5	0.11 ± 0.06	0.12 ± 0.01	0.30 ± 0.13	tr
18:3 *n*-3	L4	**0.97 ± 0.27**	**1.37 ± 0.56**	**1.84 ± 0.55**	0.10 ± 0.10
	L5	0.43 ± 0.09*	0.60 ± 0.16*	0.60 ± 0.17*	0.09 ± 0.12
20:3 *n*-6	L4	0.58 ± 0.11	0.70 ± 0.09	0.41 ± 0.08	0.52 ± 0.06
	L5	0.72 ± 0.25	0.57 ± 0.16	0.41 ± 0.06	0.49 ± 0.07
20:4 *n*-6	L4	8.59 ± 1.13	6.76 ± 1.43	14.96 ± 1.62	**11.64 ± 0.36**
	L5	9.53 ± 1.14	6.38 ± 2.62	14.96 ± 1.85	10.77 ± 0.92*
20:5 *n*-3	L4	1.56 ± 0.45	**0.55 ± 0.05**	**2.72 ± 0.56**	0.29 ± 0.11
	L5	1.18 ± 0.33	0.34 ± 0.08*	1.23 ± 0.23*	0.30 ± 0.10
22:4 *n*-6	L4	0.71 ± 0.17	1.24 ± 0.31	0.32 ± 0.06	3.88 ± 0.35
	L5	0.70 ± 0.14	1.14 ± 0.43	0.35 ± 0.05	3.35 ± 0.59
22:5 *n*-6	L4	**0.13 ± 0.02**	**0.09 ± 0.01**	**0.11 ± 0.02**	**1.97 ± 0.26**
	L5	0.17 ± 0.02*	0.12 ± 0.02*	0.18 ± 0.01*	1.52 ± 0.33*
22:5 *n*-3	L4	1.11 ± 0.22	**1.06 ± 0.17**	1.66 ± 0.18	0.42 ± 0.09
	L5	0.91 ± 0.19	0.68 ± 0.20*	1.50 ± 0.22	0.34 ± 0.07
22:6 *n*-3	L4	5.70 ± 1.15	1.98 ± 0.21	**11.42 ± 1.21**	16.55 ± 1.00
	L5	7.31 ± 1.59	2.41 ± 0.46	15.20 ± 1.82*	16.79 ± 1.70
SFA	L4	33.66 ± 3.23	**30.49 ± 3.46**	**33.10 ± 1.91**	**45.65 ± 0.50**
	L5	36.06 ± 1.25	37.37 ± 0.80*	36.61 ± 1.75*	44.58 ± 1.02*
MUFA	L4	27.79 ± 5.16	39.95 ± 4.43	19.85 ± 2.63	**17.59 ± 0.72**
	L5	26.39 ± 4.07	38.48 ± 4.44	18.08 ± 4.47	20.65 ± 2.69*
PUFA	L4	38.26 ± 2.24	**28.27 ± 0.96**	46.83 ± 1.16	36.63 ± 0.79
	L5	37.27 ± 3.18	23.48 ± 3.73*	44.99 ± 2.96	34.72 ± 2.34
LC-PUFA *n*-6	L4	10.40 ± 1.29	9.80 ± 1.88	16.23 ± 1.74	**18.01 ± 0.52**
	L5	11.43 ± 1.30	9.07 ± 3.29	16.22 ± 1.89	16.14 ± 1.50*
LC-PUFA *n*-3	L4	8.55 ± 1.58	3.87 ± 0.43	**15.93 ± 1.41**	17.28 ± 0.90
	L5	9.48 ± 1.95	3.55 ± 0.78	17.99 ± 1.76*	17.54 ± 1.59
*n*-6/*n*-3	L4	3.08 ± 0.47	4.20 ± 0.55	**1.64 ± 0.15**	**1.09 ± 0.07**
	L5	2.83 ± 0.48	4.42 ± 0.37	1.42 ± 0.06*	0.97 ± 0.12*
Δ6 desaturase	L4	3.20 ± 0.67	5.68 ± 1.67	3.38 ± 0.89	57.84 ± 3.79
	L5	4.59 ± 1.59	5.80 ± 1.73	4.22 ± 0.92	62.64 ± 9.37

Mean ± SD. Only selected fatty acids are listed, but all fatty acids analyzed were considered for percentage calculation. L4 (*n* = 7): Canola oil + 3% MFGM; L5 (*n* = 5): Canola oil + milk fat + 6% MFGM. All formulas had 0.2% AA (20:4 *n*-6) + 0.2% DHA (22:6 *n*-3). tr., traces. Δ6 desaturase calculated as the ratio 20:3 *n*-6/18:2 *n*-6.

*Indicates significant differences between groups within the same tissue (*P* < 0.05) (bold face)

## Discussion

The present study demonstrates that MFGM plus milk fat incorporation to infant formulas may increase DHA bioavailability in both plasma and tissues in suckling piglets. This provides new insights into the effects of MFGM infant formula supplementation to potentially improve neurodevelopment and anti-inflammatory properties. The higher DHA uptake seems to be related more to the transport uptake followed by polar lipids structures as PL or gangliosides across the membranes than changes in gut histology or cell proliferation.

Several reports indicate poorer cognitive development of formula-fed infants than in breastfed infants [21–23]. These differences may be related to the higher concentration of gangliosides and PL in breast milk compared with formula [24]. In fact, some animal studies suggest that individual MFGM components (PL and gangliosides) may have positive effects on both structural and functional brain development: brain growth, brain PL composition, spatial learning, and reflex development among others [25, 26]. In clinical trials, infants fed formula with added bovine MFGM scored significantly higher using the Bayley Scales of Infant and Toddler Development than infants fed with a standard formula [6, 7]. Thus, dietary supplementation with gangliosides and PL in MFGM may be a viable approach to promote brain development in formula-fed infants.

In Experiment 1 of the present study, infant formula supplemented with 3% MFGM plus milk fat (L3) increased DHA content in total fatty acids of both jejunum and liver but not in plasma. Nevertheless, LC-PUFA n-3 levels were increased in plasma total fatty acids in L2 but not in L3 respect to L1. Thus, we decided to do Experiment 2 using a higher dose of MFGM (6% MFGM + milk fat in L5 formula *vs.* 3% MFGM in L4 formula) and this corroborated not only the enhancement of DHA levels in jejunum and liver but also in plasma PL. The higher level of DHA in tissues of Experiment 1 could also be improved by higher ∆6 desaturase ratio of activity in the liver; the formula L3 with MFGM contained less ALA precursor than L2 and showed higher ∆6 desaturase ratio in liver which could have helped to increase DHA level. These results indicate that MFGM may increase plasma and tissue availability of DHA from infant formulas, and this effect may depend upon the MFGM dose used.

Milk fat (butteroil) contains PA in position beta of TAG (*sn*-2) and it is used by some companies in their formulation to avoid using palm oil (PA in *sn*-1 and *sn*-3 position) which is more attractive for food claiming. In fact, PA proportion of faeces of L1 was much higher than L3, and thus, beta-PA in milk fat contributed to higher PA uptake as reported by other authors [27, 28]. Milk fat incorporation may contribute to improve infant formula fat digestibility and to mimic breast milk PA uptake.

MFGM supplementation in early life increased serum gangliosides concentration and improved some measures of cognitive development in Chinese infants [7]. Park et al. [29] reported that dietary gangliosides may induce increase in total and relative percentage of ether PL, resulting in higher level of PUFA, specifically in ARA and DHA, compared to control animals both in PC and in PE in small intestine of weanling rats. This could contribute to the higher DHA in jejunum PL reported in the present study. MFGM has emulsifier properties similar to Tween 80 for the gut uptake evaluated using ALA labelled with stable isotopes [30]. Thus, the complex lipid structure of MFGM might improve the DHA availability for PUFA in tissues.

As villus lengths and crypt depths are overt markers of intestinal health, they were assessed to determine if intestinal histology was increased by MFGM supplementation. MFGM did not improve villi and crypts size in Experiment 1, and they behaved differently among jejunum and ileum. In fact, L2 (without palm oil) and L3 animals (with 3% MFGM and milk fat) showed the highest levels of DHA in plasma and liver and presented shorter villi than L1 formula-fed group, while there were no differences in the ileum. These results cannot confirm the previous data reported by Wu et al. [5] on better gut structure in piglets supplemented orally with saline or MFGM solution (1 g/kg body weight) during the first postnatal week and later on in piglets breastfed during the whole neonatal period until day 21. They showed higher villi length in duodenum and ileum but not in jejunum by MFGM; moreover, the crypth depth in jejunum was not different between groups and the villous/crypth ratio was not different in jejunum and ileum [5]. This experimental design was completely different to the present Experiment 1, in which piglets received a lower amount of MFGM (0.2 g/kg/day) and infant formulas with potentially less growth factors than breastfed animals, which could explain the differences between studies. In another study, Bhinder et al. [3] fed rat pups by gastric cannulas from 5 to 15 days old with either control formula, 1.2 g/L MFGM, 6 g/L MFGM or breastfed; pups displayed similar villi length in the jejunum. However, in the ileum, control formula, breastfed and 1.2 g/L MFGM pups displayed similar villus lengths, while 6 g/L MFGM supplementation resulted in significantly longer villi than in the other groups, which was surprising [3]. Crypts’ depth were shorter in control than in the rest of groups for both tissues [3]. Since the histological differences were only found with the 6 g/l MFGM dose, the rest of gut parameters were only evaluated in this group, which is an important limitation to understand the MFGM-driven effects.

On the other hand, we did not find differences in cell proliferation, evaluated for the nuclear proliferation marker Ki-67, among groups in jejunum or ileum in the Experiment 1 with an administration of 2 g/l MFGM (0.2 g/kg/day). Furthermore, no differences were found in apoptosis, evaluated by caspase-3 expression. Bhinder et al. reported in rats in both the jejunum and colon that both 6 g/l MFGM had significantly fewer positive Ki-67 cells/crypt. However, in the ileum, similar numbers of Ki-67-positive cells/crypt were recorded in all three groups [3]. These results might indicate potential benefits in cell proliferation, but they were not consistent and the 1.2 g/l MFGM dose effect was not reported.

Exogenous PL, such as PC and lyso-PC, may cross the blood–brain barrier [31]. The developing brain can synthesize and take up DHA from blood vessels and incorporate it into membrane PL (including PE, PS, and PC), resulting in enhanced neurite outgrowth, synaptogenesis, and neurogenesis [32, 33]. Surprisingly, brain tissue DHA proportion did not change because of 3% MFGM supplementation in Experiment 1, while it tended to higher levels in PL of cortex when using 6% MFGM in Experiment 2, although the differences were not statistically significant (*p* = 0.123). Moukarzel et al. reported that rat pups receiving 6 g/L of bovine MFGM supplementation in formula improved reflex development and may alter brain lipid composition, but DHA content was not different among groups in brain PE [26]. We did not analyze brain PL subclasses such as PE, PS, or PC but total PL profile, which could explain the lack of differences with respect to Moukarzel et al. [26]. We used thin-layer chromatography technique to evaluate total PL content, while other recent techniques, such as metabolomics, could even have higher sensitivity for these specific metabolites. Moreover, it is important to take into account that the brain fatty acid distribution and response to dietary lipids may differ between brain regions [34, 35]. We analyzed cortex and not the whole brain, because it was previously shown in piglets that when cerebral cortex was examined, it was responsive to both early and late dietary DHA supplementation during the postnatal phase of the brain growth spurt, while analysis of whole brain was misleading [36]. For future studies, other analysis as microarrays for brain gene expression could be of interest to demonstrate the direct effect of DHA supplementation on genes related to synaptogenesis or neurite growth processes in addition to classic behavior task evaluation.

In both experiments, the addition of MFGM to the formula resulted in less LA and especially less ALA than in the formula with same fat but without MFGM. This could be observed in Experiment 1: L2: Canola Oil vs. L3: Canola Oil + milk fat + 3% MFGM; and similarly, this also occurred in Experiment 2: L4: Canola oil + 3% MFGM vs. L5: Canola oil + milk fat + 6% MFGM). Thus, essential fatty acid precursors may not explain the higher DHA content in the tissues of L3 and L5 groups. In addition, all formulas had 0.2% AA (20:4 *n*-6) + 0.2% DHA (22:6 n-3) and Δ6 desaturase activity was similar in all tissues of both groups. This amount of ARA and DHA in the formulas ARA and DHA was used in previous studies with this animal model [20, 37, 38]. Moreover, we decided to use this low DHA and ARA dose to not saturate tissue’s DHA accretion and be able to see the effects of MFGM + milk fat if any. The ratio ARA/DHA 1/1 was set in the present study, because this was the ratio used in most commercial infant formulas when the experiments were carried out.

On the other hand, supplementation with gangliosides and MFGM in suckling rat pups may be more effective than maternal supplementation with these complex lipids during pregnancy and lactation. In fact, maternal supplementation in rats with complex milk lipids during pregnancy and lactation had a significant early impact on brain weight and ganglioside and phospholipid content in the offspring, but did not alter long-term behavioral function using standard behavioral techniques [39]. In any case, brain fatty acid composition is much more regulated than that of other peripheral tissues.

The main limitation of the present study to include was that we did not compare the results among Experiment 1 and 2, because animals from Experiment 1 were a bit younger and this affected the total time of feeding and adaptation to the formula (weight increase at day 11 of life: 8.0 ± 0.22 g/day in Experiment 1 *vs.* 6.62 ± 0.35 g/day in Experiment 2, *p* = 0.04). Nevertheless, there were not differences in animal growth within each experiment. In addition, there were some differences in the essential fatty acid composition that was lower in formulas with MFGM plus milk fat which we cannot discard that could have affect also the results. In addition, we isolated total PL fraction for tissues’ fatty acid analysis, and we cannot discard effects of experimental formulas on certain PL lipid subclasses (e.g., PC, PE, PS,…) or other brain lipid regions.

In conclusion, despite differences in the essential fatty acids between formulas, MFGM plus milk fat addition to infant formulas may increase DHA and decrease LA and ALA content in plasma and peripheral tissues, being the dose used important to achieve these effects. Nevertheless, brain DHA content was fairly regulated. PL and gangliosides structures from MFGM could contribute to emulsifier DHA from milk TAG and to increase its tissue uptake.

## Supplementary Information

Below is the link to the electronic supplementary material.

Supplementary file1 (DOCX 20 KB)

## Data Availability

The data presented in this study are available on reasonable request from the corresponding author.
