# Supplementary material for: Milk fat globule membrane plus milk fat increase docosahexaenoic acid availability in infant formulas
Source: Eur J Nutr. 2022 Oct 25;62(2):833–45. doi: 10.1007/s00394-022-03024-5 (PMC9941230; doi:10.1007/s00394-022-03024-5)
Supplement: Supplementary file 1 — Supplementary file1 (DOCX 20 KB) [file 394_2022_3024_MOESM1_ESM.docx]

Supplemental Table 1. ArachidSonic (AA) and docosahexaenoic acid (DHA) concentrations in lipid fractions of piglets from Experiment 1 and Experiment 2.

| Experiment 1 | | | |  | Experiment 2 | | | |
| --- | --- | --- | --- | --- | --- | --- | --- | --- |
| mg/g |  | Jejunum | Cortex^†^ |  |  | Jejunum | Colon | Cortex^†^ |
| Phospholipids | | |  |  |  |  |  |  |
| AA | L1 | 0.29 ± 0.07 | 2.53 ± 0.42 |  | L4 | 0.31 ± 0.06 | 0.14 ± 0.01 | 2.43 ± 0.24 |
|  | L2 | 0.25 ± 0.09 | 2.42 ± 0.17 |  | L5 | 0.35 ± 0.14 | 0.17 ± 0.03 | 2.35 ± 0.31 |
|  | L3 | 0.27 ± 0.04 | 2.51 ± 0.09 |  |  |  |  |  |
| DHA | L1 | 0.20 ± 0.07 | 3.41 ± 0.60 |  | L4 | 0.23 ± 0.06 | **0.03 ± 0.01** | 3.70 ± 0.35 |
|  | L2 | 0.18 ± 0.02 | 3.51 ± 0.26 |  | L5 | 0.30 ± 0.14 | **0.05 ± 0.01*** | 3.94 ± 0.51 |
|  | L3 | 0.21 ± 0.05 | 3.49 ± 0.30 |  |  |  |  |  |
| Triacylglycerols | | |  |  |  |  |  |  |
| AA | L1 | 0.01 ± 0.01 |  |  | L4 | 0.01 ± 0.01 | 0.01 ± 0.01 |  |
|  | L2 | 0.01 ± 0.01 |  |  | L5 | 0.01 ± 0.01 | 0.01 ± 0.01 |  |
|  | L3 | 0.01 ± 0.01 |  |  |  |  |  |  |
| DHA | L1 | 0.01 ± 0.01 |  |  | L4 | 0.01 ± 0.01 | 0.02 ± 0.01 |  |
|  | L2 | 0.01 ± 0.01 |  |  | L5 | 0.01 ± 0.01 | 0.02 ± 0.02 |  |
|  | L3 | 0.01 ± 0.01 |  |  |  |  |  |  |
| Cholesteryl esters | | |  |  |  |  |  |  |
| AA | L1 | 0.01 ± 0.01 |  |  | L4 | 0.01 ± 0.01 | 0.01 ± 0.01 |  |
|  | L2 | 0.01 ± 0.01 |  |  | L5 | 0.01 ± 0.01 | 0.01 ± 0.01 |  |
|  | L3 | 0.01 ± 0.01 |  |  |  |  |  |  |
| DHA | L1 | 0.01 ± 0.01 |  |  | L4 | tr. | tr. |  |
|  | L2 | 0.01 ± 0.01 |  |  | L5 | tr. | tr. |  |
|  | L3 | 0.01 ± 0.01 |  |  |  |  |  |  |
| Non-esterified fatty acids | | |  |  |  |  |  |  |
| AA | L1 | 0.02 ± 0.01 |  |  | L4 | 0.13 ± 0.06 | 0.14 ± 0.03 |  |
|  | L2 | 0.02 ± 0.01 |  |  | L5 | 0.11 ± 0.06 | 0.12 ± 0.05 |  |
|  | L3 | 0.02 ± 0.01 |  |  |  |  |  |  |
| DHA | L1 | 0.01 ± 0.01 |  |  | L4 | 0.07 ± 0.03 | 0.04 ± 0.01 |  |
|  | L2 | 0.01 ± 0.01 |  |  | L5 | 0.06 ± 0.04 | 0.04 ± 0.02 |  |
|  | L3 | 0.01 ± 0.01 |  |  |  |  |  |  |

Data are mean ± SD. L1 (n=8): vegetal fat (with palm oil); L2 (n=8): Canola Oil; L3 (n=8): Canola Oil + milk fat + 3% MFGM; L4 (n=7): Canola oil + 3% MFGM; L5 (n=5): Canola oil + milk fat + 6% MFGM. All formulas had 0.2% AA (20:4 n-6) + 0.2% DHA (22:6 n-3). tr., traces. ^†^Only phospholipid fraction was analyzed for fatty acid profile since it accounts for more than 98% of total fatty acids for brain. Values not sharing the same superscript letters within a tissue of Experiment 1 indicate statistical differences between groups (P < 0.05).*Indicates significant differences between groups within the same tissue in Experiment 2 (P < 0.05).
